# Capturing the Influence of Jet Fluctuations on Particles in Plasma Spraying

**DOI:** 10.1007/s11666-021-01307-7

**Published:** 2022-01-18

**Authors:** K. Bobzin, H. Heinemann, A. O’Brien

**Affiliations:** Surface Engineering Institute, RWTH Aachen University, Kackertstr. 15, 52072 Aachen, Germany

**Keywords:** Abel transformation, CFD simulation, high-speed imaging, plasma spraying, process stability, python

## Abstract

Instabilities and fluctuations of the plasma jet in a thermal spray process can have a significant influence on the particle in-flight temperatures and velocities, affecting the properties of resulting plasma-sprayed coatings. Presented in this paper is a novel method for capturing the effects particles are exposed to in the plasma spraying process. High-speed camera images of a plasma jet generated by a cascaded three-cathode plasma generator (TriplexPro-210) were recorded for varying operating conditions. The images are processed using the inverse Abel transform. This transformation accounts for the fact that the images represent a 2D projection of the 3D jet and generates more accurate intensity values that the sprayed particles would experience. These images are then combined with particle tracks resulting from CFD simulations of the plasma jet to match the particles path with the recorded plasma jet. This new method allows a precise description of the plasma intensity experienced by individual particles with a high temporal resolution. The results show a high sensitivity of the method, even detecting the influence on the particles of the plasma jet originating from the cascaded triple arc plasma generator, which is considered as rather stable.

## Introduction

First developed in the 1910s and 1920s (Ref 1), thermal spraying is a coating process in which particles are deposited onto a substrate in molten, semi-molten or solid state. With this technique, coatings ranging in thickness from microns to millimeters can be applied over a large area and at a fast deposition rate compared to other similar methods. Thermal spraying can be implemented to deposit a broad range of materials, from single-phase materials such as metals, ceramics and polymers, to composite materials and functionally gradient materials. The specific material(s) chosen are mainly dependent on the desired function of the coating, allowing for application-specific tailoring of the coating to withstand chemical/mechanical attack, be a thermal barrier, act as functional coatings or for aesthetic effects. This has allowed thermal spray coatings to find application in several areas in industry, including transport, energy and manufacturing. In particular, thermal-sprayed coatings have been used in advanced gas turbine components which has driven much of the research (Ref 2).

Plasma spraying is a thermal spray technique that makes use of a plasma jet to heat and simultaneously accelerate the feedstock (generally a powder) (Ref 3). The process exhibits very high temperatures, with plasma jet temperatures reaching as high as 20,000 K (Ref 4), allowing the processing of the wide range materials mentioned above. The primary limit to this technique is that the melting temperature of the coating material must be somewhat 300 K below the vaporization (or decomposition) temperature in order to avoid low deposition efficiencies (Ref 5).

A major issue in describing this process is the large number of interactions that would need to be described to capture each step in this process: the electron–gas interactions that create the plasma, the plasma–particle interactions when the feedstock is introduced to the plasma jet and finally the particle–substrate interactions when the coating material impacts on the substrate (Ref 6). There are inherent fluctuations from step one of this process, as there are instabilities in the arc that creates the plasma initially. This arc instability causes variation in the input electrical power as well as pressure fluctuations (Ref 7) whose effect is only compounded by turbulence in the plasma flow. These fluctuations in the plasma jet cause residence times to vary between particles and can result in uneven heating of the feedstock which can have significant implications on the properties of the resulting coatings (Ref 8).

One approach used to improve the stability of the jet has been the introduction of multiple arcs and the cascaded anode design. The cascaded design of the anode increases the distance between the electrodes thus raising the arc voltage rather than the arc current in order to achieve the necessary power. At the same time, the movement of the arc is restricted, which also reduces the fluctuation of the power output. By combining this measure with the division into three arcs, the stability of the power output can be further increased, resulting in a very stable plasma jet (Ref 9, 10).

However, even this rather stable plasma jet is affected by turbulence and thus continues to exhibit fluctuations. These varying jet sizes and movements affect the temperatures experienced by the individual particles as they pass through the plasma. An approach to generally describe the instability of plasma jets, including observations of fluctuations in the electric arc and the effect this has on the plasma jet and resulting coatings, has already been performed in literature (Ref 11, 12). However, until now they did not concentrate on the instabilities in the jet of the cascaded three-cathode plasma generator.

Advances in camera hardware have allowed for new optical approaches to describe the plasma spraying process. Many of these focus on the particles in flight after they have passed through the plasma jet (Ref 13, 14). It is likely that optical approaches will continue to improve in the future with the abilities of cameras constantly improving (Ref 15). State-of-the-art cameras are now able to precisely capture the highly dynamic processes of turbulence in the plasma free jet. In a recent paper by the authors, it has already been shown that the method of using a high-speed camera is able to assess the stability of a plasma jet in plasma spraying (Ref 16).

The approach of this paper was to now apply a high-speed camera to capture the plasma jet fluctuations and its possible influence on the particles in plasma spraying. This was carried out on the assumed stable cascaded multi cathode plasma generator, to evaluate whether this type of plasma generator can still lead to varying particle properties. Attempting to follow individual particles’ flight paths as they pass through the plasma jet presents some unique challenges however. The extreme brightness of the plasma makes it impossible to follow the particle flight paths using standalone high-speed imaging. For this reason, a previously developed CFD simulation was used to calculate the particle trajectories within the plasma jet. These trajectories were then mapped onto the high-speed recordings of the jet. By combining the recordings and the particle tracks, the possible effect of the plasma jet fluctuations on individual particles was assessed.

## Experimental Procedure

### High-Speed Imaging of Plasma Jet

The cascaded triple arc plasma generator used in this study is the TriplexPro-210 Plasma Spray Gun (Oerlikon Metco, Pfäffikon, Switzerland). The torch was equipped with a 9 mm diameter nozzle. As mentioned earlier, this plasma gun exhibits an increased plasma stability compared to commonly used non-cascaded single-arc models like the F4 or SG-100. Pure argon was used as plasma gas. The electric current was varied at the levels *I* = 200, 350 and 500 A, while the plasma gas flow rate took the following steps $\dot{V}_{{{\text{gas}}}}$ = 50, 70, 120 slpm. The combinations of these lead to the process parameters outlined in Table 1.

**Table 1:** Process parameters

	Arc current *I*, A	Gas flow rate $\dot{V}_{{{\text{gas}}}}$, slpm
Trial 1	200	50
Trial 2	200	70
Trial 3	200	120
Trial 4	350	50
Trial 5	350	70
Trial 6	350	120
Trial 7	500	50
Trial 8	500	70
Trial 9	500	120

**Fig. 1:**
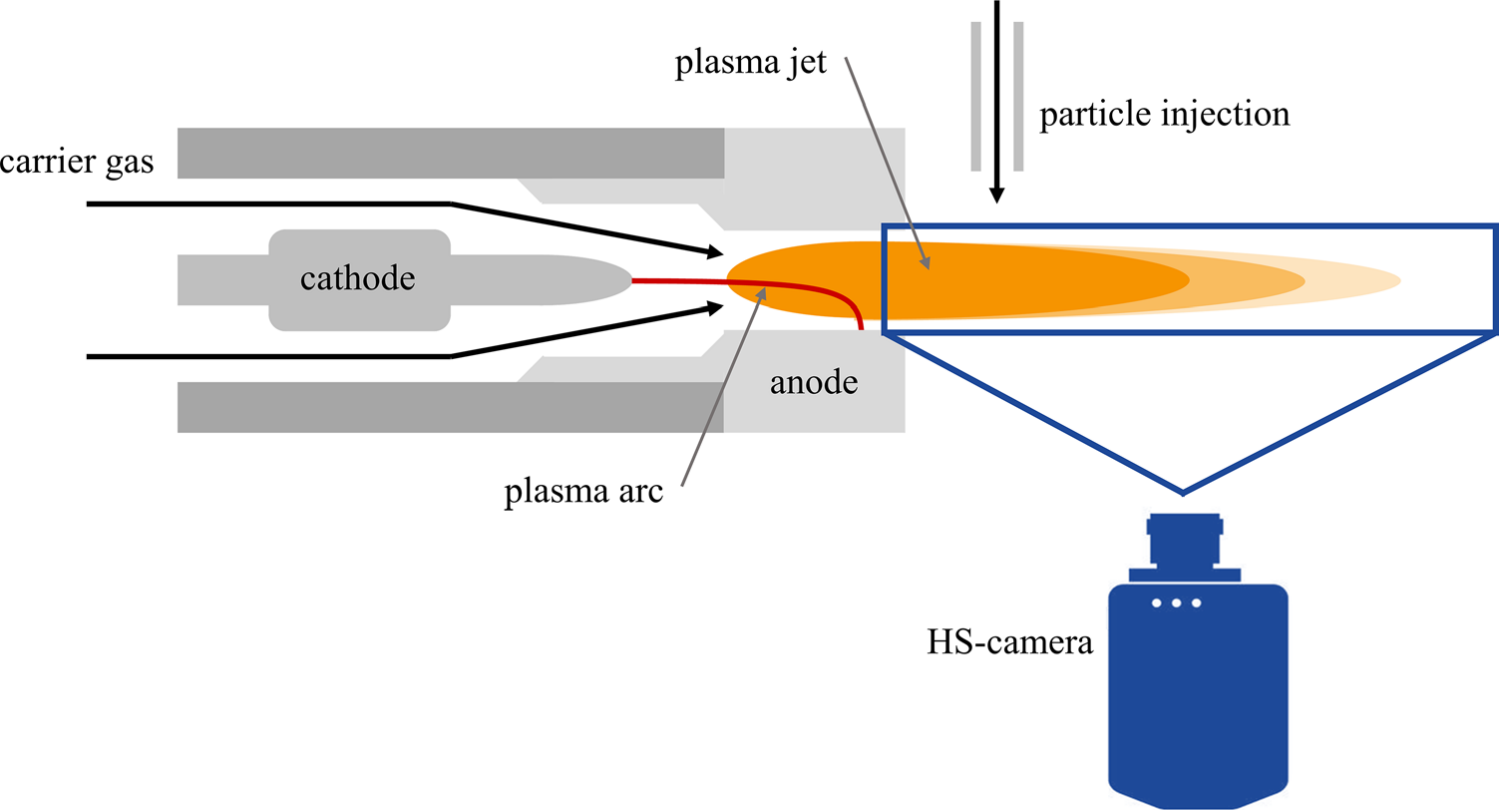
Experimental setup for plasma torch and high-speed camera

High-speed videos of the plasma jet emerging from the plasma generator were recorded perpendicular to the torch axis. The camera used is a Fastcam model SA-Z by Photron, which was mounted with an Irix 150 mm f/2.38 macro-lens. Higher frame rates can only be achieved with the Photron Fastcam by reducing the maximum image size and thus the field of view. For this investigation, a frame rate of 210,000 fps was used, along with an exposure time of 1.25 μs. This allowed for a resolution of 384 × 160 pixels (in real terms approx. 70 × 30 mm), resulting in a field of view large enough to capture the entire length of the plasma jet while maximizing the frame rate. A neutral density filter (ND64) was used to decrease the intensity of the recorded imaging to 1.5% of the original, preventing overexposure. An additional ultraviolet filter was also used as a customary protection of the lens. During this analysis, the powder feeding was deactivated. For clarity, the complete experimental setup with plasma torch and high-speed camera is illustrated in Fig. 1.

### Calculation of the Particle Trajectories

**Fig. 2:**
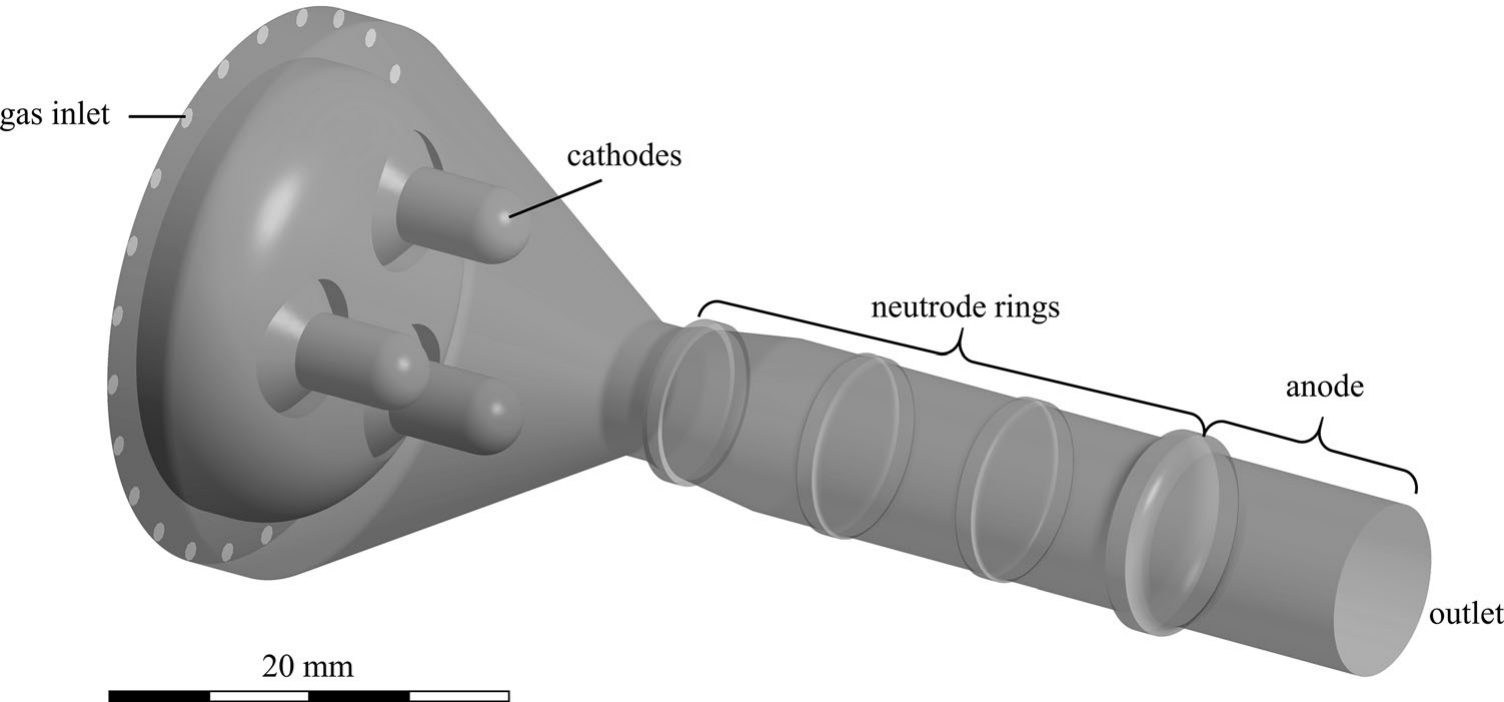
Schematic representation of the plasma generator model

**Fig. 3:**
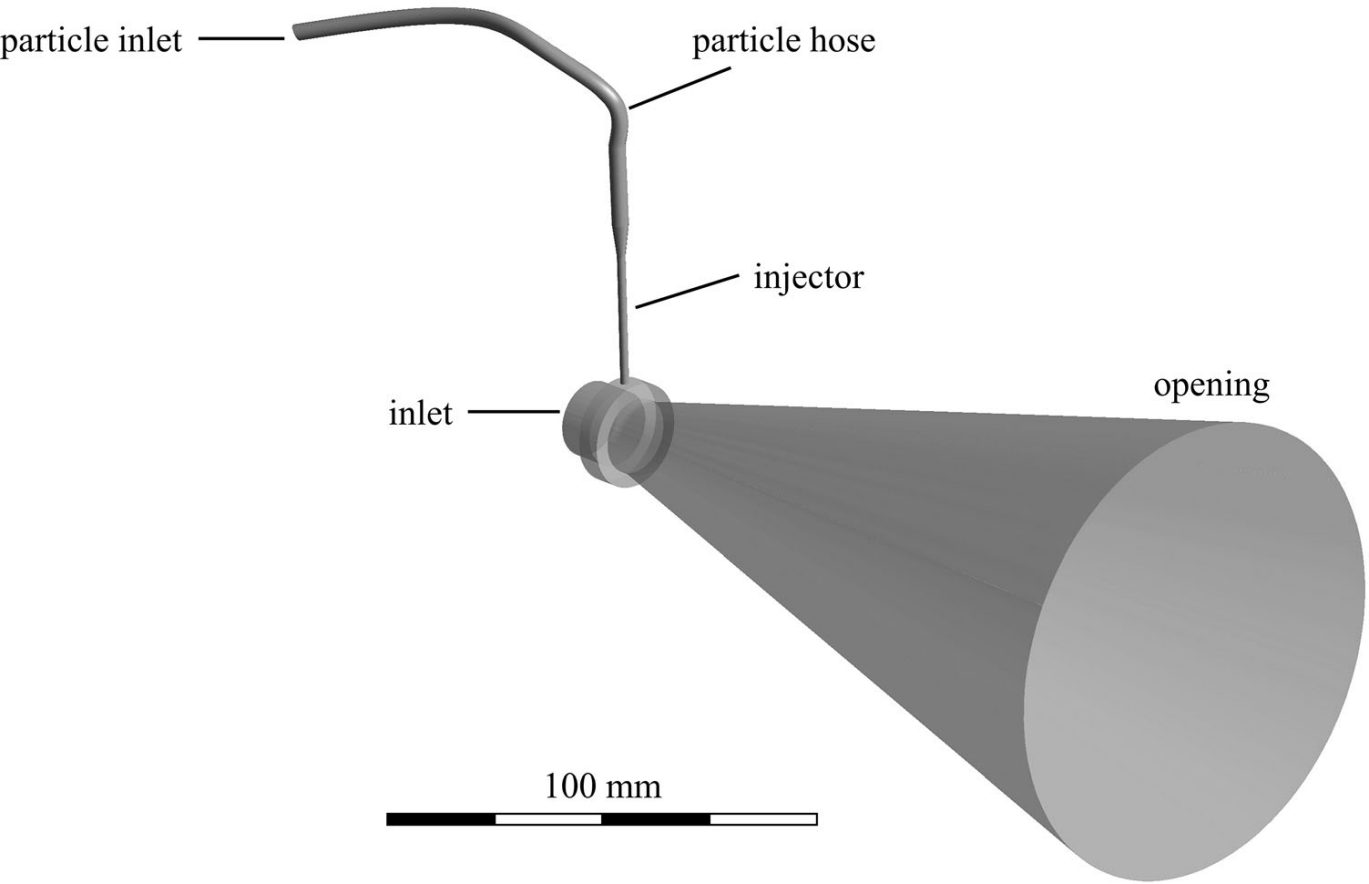
Representation of the model to calculate the particle trajectories in the plasma jet

The particle trajectories were calculated using an existing CFD simulation in Ansys CFX. The simulations are composed of two different steady-state models. The first model shown in Fig. 2 solves the magnet-hydrodynamic equations within the plasma generator. The model exploits the axial symmetry of the generator shown and solves the equations only in 120°, i.e., exactly one-third of the geometry. In between are planes that periodically continue the boundary conditions. The mesh consists of 885,629 elements, which are composed of tetrahedra, pyramids, wedges and hexahedra. The edge length of these elements is within the range of 0.1 to 0.3 mm. Basically, the model simulates the plasma as a fluid with the properties of a plasma. In the model, the electric current is imposed on the cathodes and leads to a corresponding heating and acceleration of the plasma gas in the generator. The model was firstly developed in 2011 (Ref 17) and has been consequently expanded (Ref 18, 19). The approach is capable of calculating the velocity and temperature profile of the plasma gas at the outlet of a TriplexPro-210 torch for the given process parameters. This temperature and velocity profiles are then used as an input for the second model, which calculates the propagation of the plasma jet into the atmosphere as well as the acceleration and melting behavior of injected particles using a Lagrangian particle tracking approach. The model setup is shown in Fig. 3, the particles and the corresponding injector gas are injected into the injector hose. At the main inlet, the former mentioned plasma gas temperatures and velocities of the plasma generator model are used as a boundary condition. In the model domain, 1000 are particles injected via the injector into the plasma jet and its acceleration and heating behavior are calculated. Alumina particles are simulated with a mean particle diameter of *d*_p_ = 37.7 µm and standard deviation of *σ*_p_ = 11 µm, representing the particle size distribution − 45 +22 µm of the commercially available feedstock AMDRY 6062 (Oerlikon Metco, Pfäffikon, Switzerland). The trajectories of the particles are calculated using a steady-state approach. The gas flow is calculated by assuming a turbulent flow, describing the turbulences using the shear-stress-transport model. The particle velocities are calculated using the following drag coefficient (*C*_D_) correlation:

$$C_{{\text{D}}} = \frac{24}{{{\text{Re}}_{{\text{p}}} }}, \quad {\text{Re}}_{{\text{p}}} \le 0.2$$

$$C_{{\text{D}}} = \frac{24}{{{\text{Re}}_{{\text{p}}} }} \left( {1 + 0.1{\text{Re}}_{{\text{p}}}^{0.99} } \right),\quad 0.2 < {\text{Re}}_{{\text{p}}} \le 2$$

$$C_{{\text{D}}} = \frac{24}{{{\text{Re}}_{{\text{p}}} }} \left( {1 + 0.11{\text{Re}}_{{\text{p}}}^{0.81} } \right), \quad 2 < {\text{Re}}_{{\text{p}}} \le 21$$

$$C_{{\text{D}}} = \frac{24}{{{\text{Re}}_{{\text{p}}} }} \left( {1 + 0.189{\text{Re}}_{{\text{p}}}^{0.62} } \right),\quad 21 < {\text{Re}}_{{\text{p}}} \le 500$$

$$C_{{\text{D}}} = 0.44, \quad 500 < {\text{Re}}_{{\text{p}}} .$$

The shown *C*_D_ values are depending on the particles Reynolds number Re_p_ following the relations proposed in (Ref 20-22). In this context, Re_p_ denotes the Reynolds number of the particles, being a function of the injector gas density *ρ*_g_ and dynamic viscosity *µ*_g_, the particles’ diameter *d*_p_ and the slip velocity between the particle and the injector gas (*v*_g_–*v*_p_):

$${\text{Re}}_{{\text{p}}} = \frac{{\rho_{{\text{g}}} \left( {v_{{\text{g}}} {-}v_{{\text{p}}} } \right)d_{{\text{p}}} }}{{\mu_{{\text{g}}} }}.$$

In this model, the particles are assumed to be one-dimensional solid particle. The heat transfer and the melting degree are calculated using subroutines in ANSYS. Yet these processes are not described in detail as they are not relevant for the particle’s trajectories. The used mesh consists of 2,363,272 elements, using tetrahedra, pyramids, wedges and hexahedra as element types which exhibit an edge length ranging between 0.3 and 2 mm. Further details and more information of this model can be found in (Ref 23). Both models have been validated by measuring the temperatures of the plasma jet experimentally using computer tomographic, likewise the second model was validated by measuring the particle temperatures, velocities and positions in the plasma jet and comparing the particles trajectories with high-speed images of the injection region (Ref 24). In combination, the two models are able to predict the particle trajectories of particles for the TriplexPro-210 plasma generator.

In contrast to the recorded images, the particle trajectories were calculated only for one process parameter. The reason for this is that the method is novel and in this first step we only wanted to investigate the possible influence of different process parameters on the particle properties. If the process parameters, current and gas flow were also varied in the simulations, the injector gas flow would also need to be adjusted. This would create a chain of interdependencies that would be difficult to trace. In order to not affect the comparability of the presented results, only the process parameters of the recorded images were varied.

**Fig. 4:**
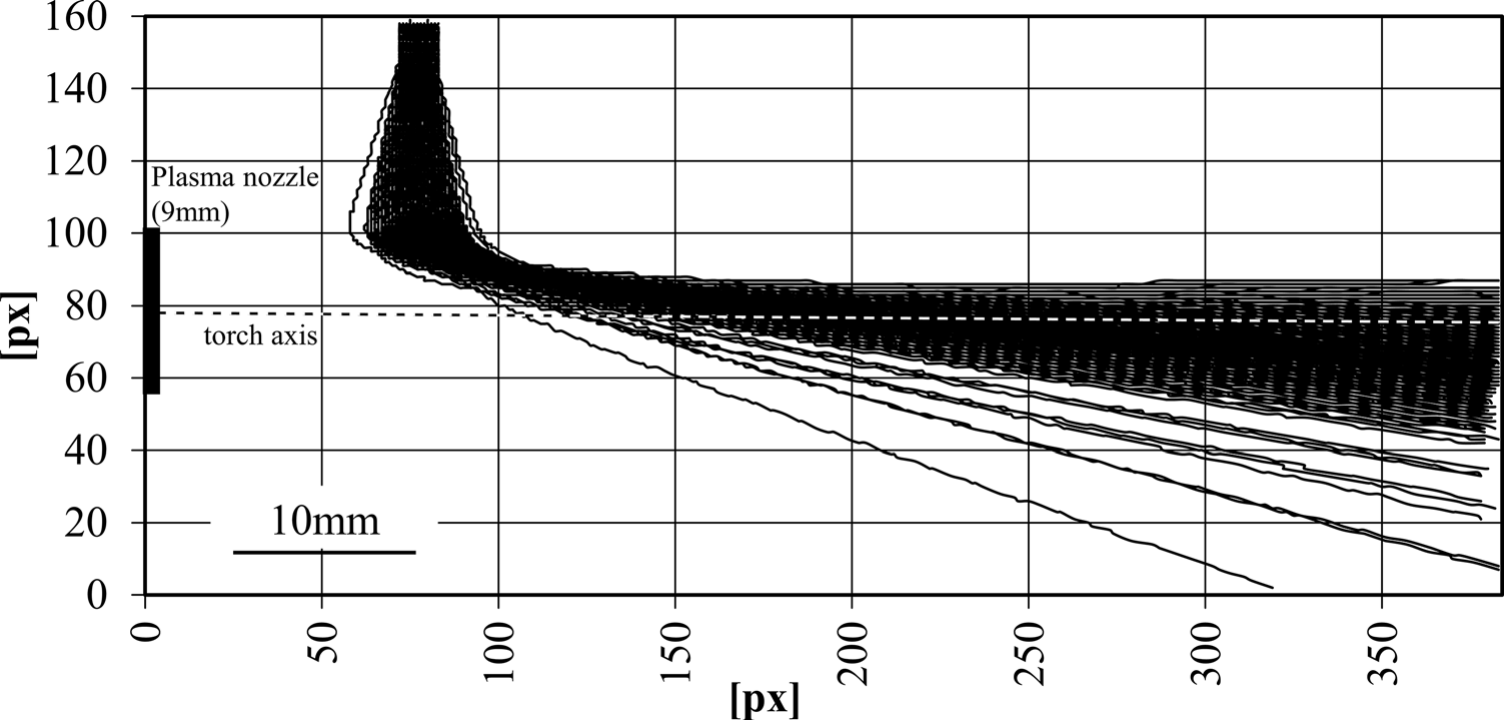
Particle flight paths imposed over field of view of high-speed plasma jet images

A current of *I* = 500 A and a plasma gas flow rate of $\dot{V}_{{{\text{gas}}}}$ = 50 slpm were used for the calculations in the plasma generator model. A powder feed rate of $\dot{m}_{{\text{p}}}$ = 24 g/min and an injector gas flow rate of $\dot{V}_{{{\text{inj}}}}$ = 5 slpm were applied as process parameters. The outcome of the model is individual 1000 particle trajectories, each containing the *x*-, *y*- and *z*- components for the particles’ location and the respective time. Since the camera images only have two dimensions, the particles were projected onto a plane that lies along the axis of the injector and the plasma torch, thus reducing the dimensions as well. Figure 4 shows the calculated particle trajectories projected onto the previously mentioned plane and displayed in the field of view of the recorded high-speed camera images.

### Image Processing (Python/Matlab)

An inverse Abel transformation, as shown in the equation below, was carried out on the high-speed camera images in order to correct for the fact that they are a 2D projection of the plasma jet.

$$f\left( {r, z} \right) = - \frac{1}{\pi }\mathop \smallint \limits_{r}^{\infty } \frac{{{\text{d}}F\left( {r,z} \right)}}{{{\text{d}}y}}\frac{{{\text{d}}y}}{{\sqrt {y^{2} - r^{2} } }}.$$

It is necessary to apply this transformation to the plasma jet images before the mapping can be done. This is because the camera captures a 2D projection of an originally 3D plasma jet. The inverse Abel transform restores the 3D object from its 2D projection. If this transformation would not be applied the captured intensities of the plasma jet would not correctly represent the reality. The transformation was done in Python, using a package called PyAbel (Ref 25). This package provides efficient implementation of several Abel transform algorithms, allowing for quick trial of different algorithms in order to determine optimal results in reasonable time.

**Fig. 5:**
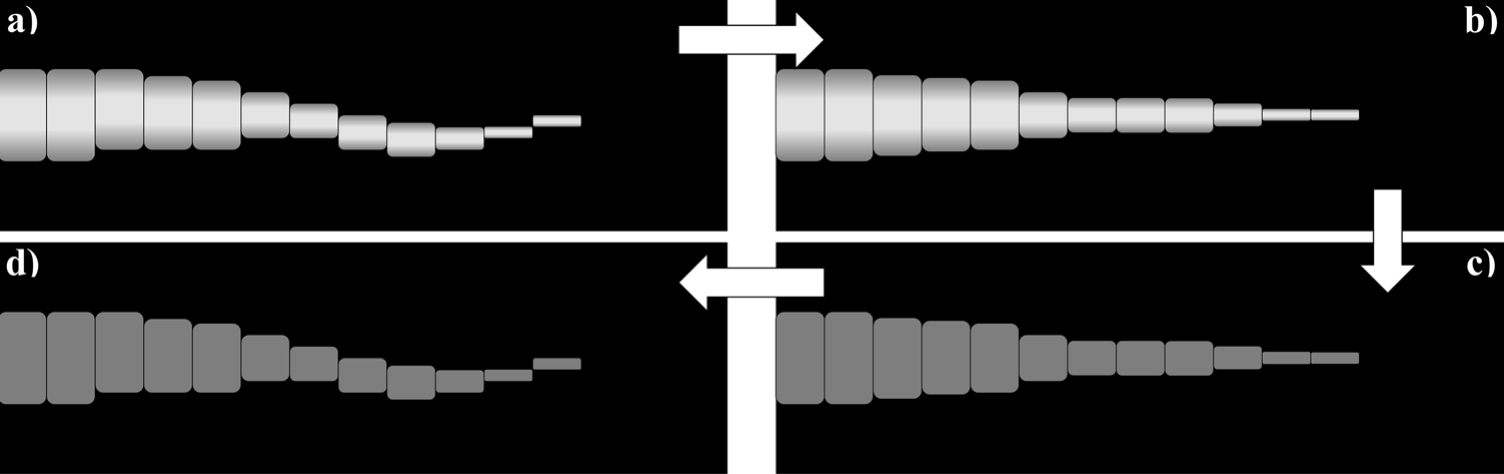
Inverse Abel transform process. (a) Original image, (b) central symmetry applied, (c) inverse Abel transform applied and (d) central symmetry reversed

The instability of the plasma jet makes carrying out the transform difficult, as a prerequisite to carry it out is cylindrical symmetry. In order to overcome this issue, the Abel inversion is applied individually on the columns of the images using its individual center of intensity. The complete effect of this pre-processing is illustrated in Fig. 5. In a first step, each image loaded into Python is split into pixel columns (Fig. 5a). These columns are then individually centered by their intensity as shown in using the SciPy center_of_mass function. SciPy is an open-source Python-based library that has been optimized for scientific computing and allows for easy to read and efficient code. As a result, it has established itself as commonly used library scientists and engineers to solve data problems (Ref 26). The single-pixel columns were then re-joined into a single image as shown in Fig. 5(b). This was achieved with the numpy split, roll and concatenate functions outlined below. In these formulas, r contains the SciPy center_of_mass data to center the jet in each image.

$$\begin{aligned} & Z\_s = np.split\left( {Z, \, np.size\left( {original, \, axis = 1} \right), \, axis = 1} \right) \\ & {\text{for}}\;i\;{\text{in}}\;{\text{range}}\left( {np.size\left( {Z, \, axis = 1} \right)} \right): \\ & Z\_s\left[ i \right] = np.roll\left( {Z\_s\left[ i \right], \, - r\left[ i \right]} \right) \\ & Z = np.concatenate\left( {Z\_s, \, axis = 1} \right) \\ \end{aligned}$$

The inverse Abel transformation is then applied to the image as shown in Fig. 5(c). The idea is to minimize the impact flickering of the jet has on one transform. This shift of the images must then be reversed after the transform has been carried out, as the particles would experience this flickering should the flight paths intersect. The back-shifting process is shown in Fig. 5d). The complete parameters to carry out the actual transformation are outlined below.

$$\begin{aligned} & Z\_inverse \, = \, (abel.Transform(Z, \, direction = ^{\prime}inverse^{\prime}, \, method = ^{\prime}hansenlaw^{\prime}, \\ & symmetry\_axis = 0, \, symmetrize\_method = ^{\prime}average^{\prime}, \\ & use\_quadrants = \left( {True,True,True,True} \right) \\ & ).transform) \\ \end{aligned}$$

Due to the extremely high frame rate used in this study, the number of files is in the thousands, and so an efficient algorithm is advantageous. A thorough investigation of the different transform algorithm methods is already available (Ref 27), both for sensitivity and speed, and it was found that the iterative Hansen Law method provides optimal results (Ref 28). The *direction* was set to inverse as we wish to apply the inverse Abel transform to the 2D projection of the plasma jet that is our image. The *symmetry_axis* is used to specify in your image which axis is the symmetrical axis around which to apply the transformation, 0 being the vertical axis which we need. The *use_quadrants* variables can be adjusted to take only specific quadrants of the image into account and to adjust when data quality is poor/obscured in part of the image. As we wanted to make use of all the recorded data, this was all set to True. The *symmetrize_method* can be set to “average” or “Fourier.” It was not found that “Fourier” resulted in improved results, so the default method “average” was applied.

### Mapping of the particle trajectories onto the high-speed images

**Fig. 6:**
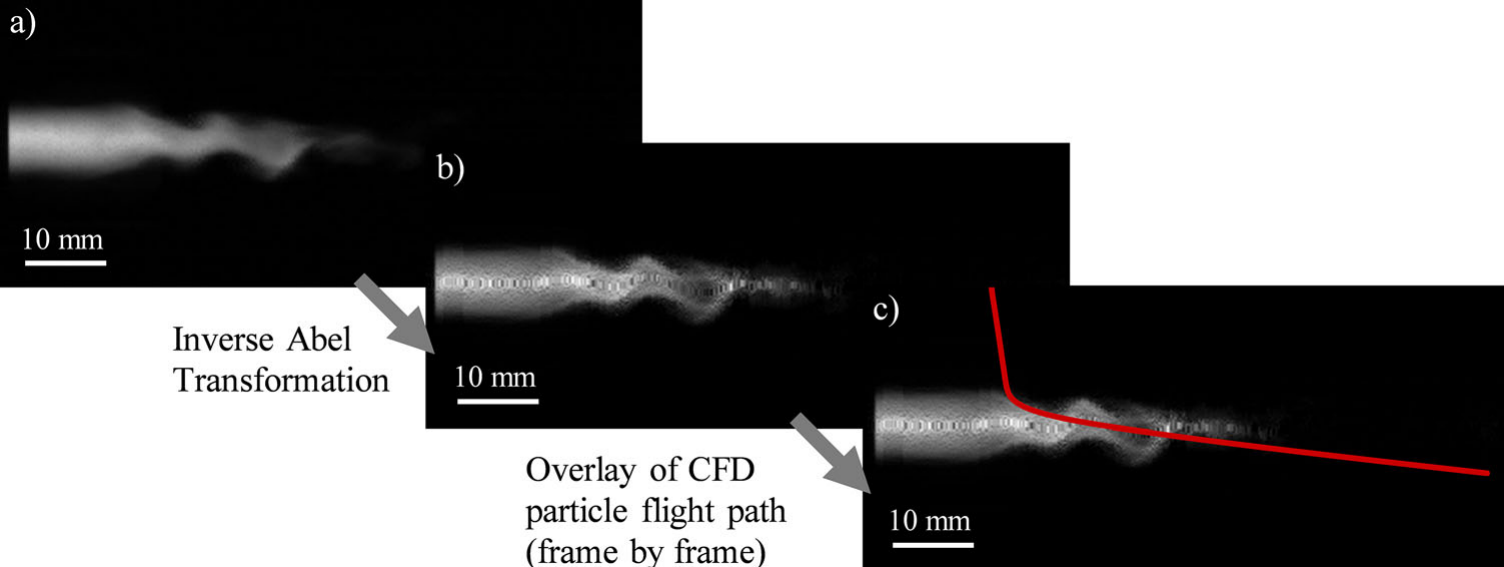
(a) Original plasma jet image for an Argon plasma gas flow rate of $\dot{V}_{{{\text{gas}}}}$ = 50 slpm and an electrical current of *I* = 500 A; (b) inverse Abel transformation of the plasma jet image; and (c) calculated particle trajectory mapped on plasma image

The calculated particle trajectories and high-speed imaging can then be combined in order to determine the potential plasma intensities profile single particles would experience. A full illustration of the complete image processing method carried out can be seen in Fig. 6. In Fig. 6(a), the original image recorded by the high-speed camera is again shown. Figure 6(b) illustrates this image after the inverse Abel transform has been applied. Figure 6(c) then overlays a sample particle flight path over this image. In order to find the time-resolved intensity profile experienced by the particles as they travel along their flight path, it is not possible to simply overlay the flight path over one image. The time steps from the CFD simulation were converted to equivalent image frames and for each step the intensity at each particle position in space could then be read. This was then combined to detail the time-resolved intensity profile experienced by each particle.

It must be noted that the CFD simulation has a longer flight path than the trajectory presented in Fig. 6c). This is due to the reason that the simulation includes a representation of the powder hose and the powder injector. The path and consequently the needed time of a single particle to propagate through this section is very large in relation to the length of the recordings. If one were to take the uncorrected simulation time as a basis, the particles would only arrive in the interesting section after the end of the video. For this reason, the frame numbers were shifted after assessing the intensity profile such that each particle enters the image on frame 1 (globally this is a different frame for each particle). This was done to simplify a comparison of the times the particles take to reach the plasma jet, residence times, etc.

## Results and Discussion

**Fig. 7:**
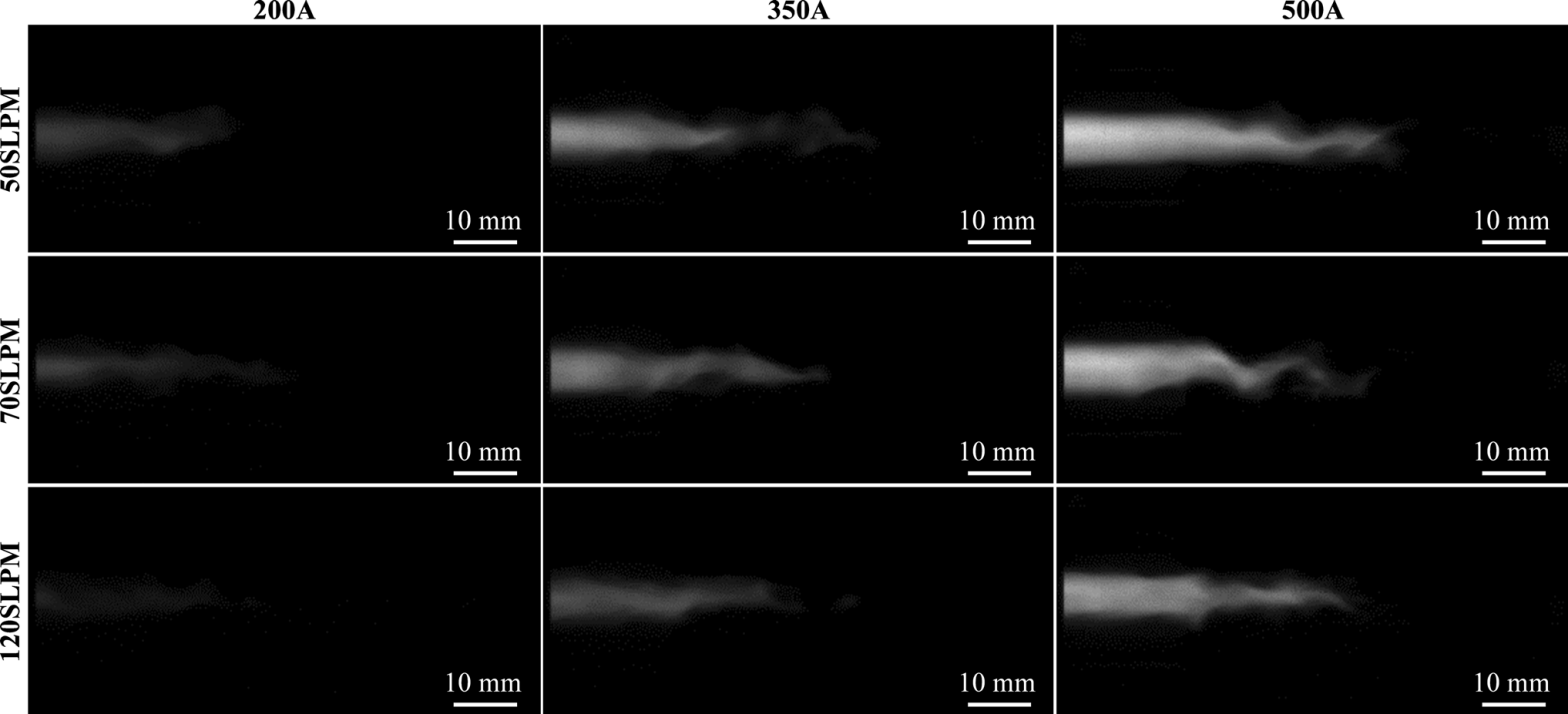
High-speed camera images of plasma jet under different current and gas flow parameters

A sample of the plasma jet images for each of the arc current and gas flow parameters is shown in Fig. 7. The plasma jet shown in the top left has the smallest arc current and plasma gas flow, at *I* = 200 A and $\dot{V}_{{{\text{gas}}}}$ = 50 slpm, respectively, while the plasma jet in the bottom right has the highest at *I* = 500 A and $\dot{V}_{{{\text{gas}}}}$ = 120 slpm. Comparing the images from left to right, higher arc currents in the creation of the plasma flame result in a greater emission intensity. This was to be expected, since higher currents also lead to higher power levels. At the same time, the emission intensity decreases with increasing plasma gas flow rate. This influence was again predictable and can be explained by the fact that with higher gas flow rates a greater mass has to be heated and thus the plasma jet reaches lower temperatures. The images taken of the parameters with a current of *I* = 200 A barely show a plasma jet at all. This is due to the deliberately chosen consistent parameters during recording, which lead to a strong attenuation of the images’ brightness. This is especially true for the higher gas flow rate. Therefore, the results of these parameters are omitted in the following.

**Fig. 8:**
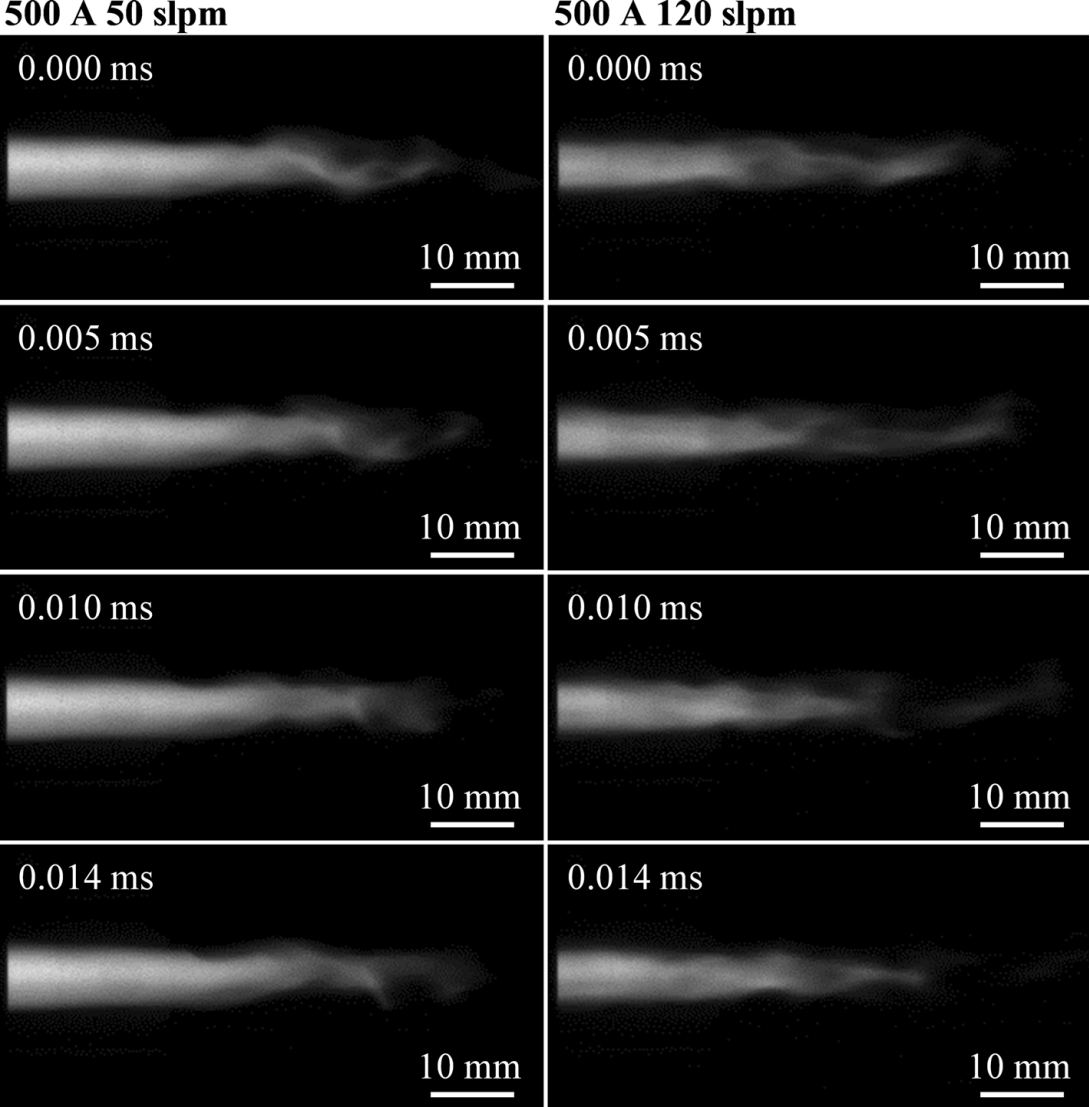
Consecutive frames of the plasma jet at a current of *I* = 500 A at two different gas flow rates $\dot{V}_{{{\text{gas}}}}$ = 50 slpm and $\dot{V}_{{{\text{gas}}}}$ = 120 slpm

Figure 8 shows the development of the plasma jet over the course of 14 ms. Two parameters, both with a current of *I* = 500 A and two different gas flow rates $\dot{V}_{{{\text{gas}}}}$ = 50 slpm and $\dot{V}_{{{\text{gas}}}}$ = 120 slpm are displayed. This is supplemented with videos of the jet corresponding to the above parameters, accessible in the supplementary material Part 1+2, which indicate a difference between parameters. However, it is difficult to evaluate this difference. Purely visually it is not possible to tell which one of the parameters exhibits a higher stability. The same applies to the photographs shown here; the sequence of images alone does not reveal any significant difference between the parameters. This highlights the need for the methodical approach toward a quantitative assessment. The results of this approach are presented onward.

**Fig. 9:**
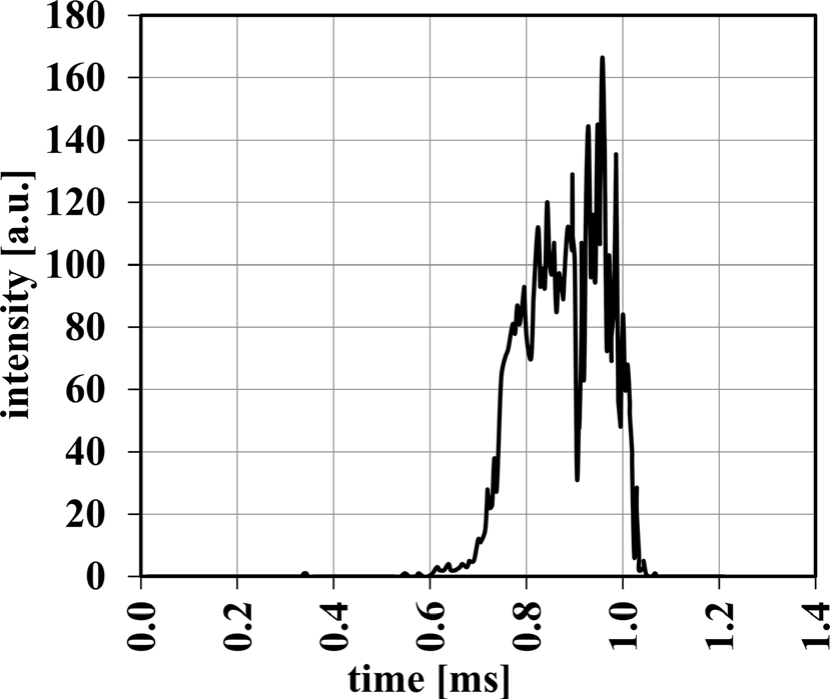
Typical experienced particle intensity profile (*I* = 500 A, $\dot{V}_{{{\text{gas}}}}$ = 120 slpm)

As mentioned, 1000 particles were included in the CFD simulation. The intensity profiles for each of these particles were calculated for different plasma gas flows and arc currents. A typical example of the intensity profile of a single particle of trial 9 (*I* = 500 A, $\dot{V}_{{{\text{gas}}}}$ = 120 slpm) is shown in Fig. 9, an animation of which can be found in the supplementary material (Part 3). For the first approximately 0.6 ms, the particle does not come in contact with the plasma jet. At this point, it was moving slowly and was not exposed to significantly increased temperatures of the plasma jet. At about *t* = 0.6 ms, the intensity begins to increase and only little fluctuations can be observed. These variations could be caused by the inherent fluctuations in the plasma jet, probably resulting from instabilities in the electric arc. The observable fluctuations become greater as the particle continues its flight, as turbulence impacts the jet more and more. Eventually, the particle exits the jet completely and continues out of frame. It must be remembered that the Triplex plasma gun used is considered to be a stable plasma jet and the particle only stayed in the apparent plasma jet for about *t* = 0.4 ms. However, as made clear in Fig. 9 even during this short period of time, the intensity fluctuations experienced by a single particle can be extreme, which can drastically alter the in-flight properties of the particles like its temperature.

**Fig. 10:**
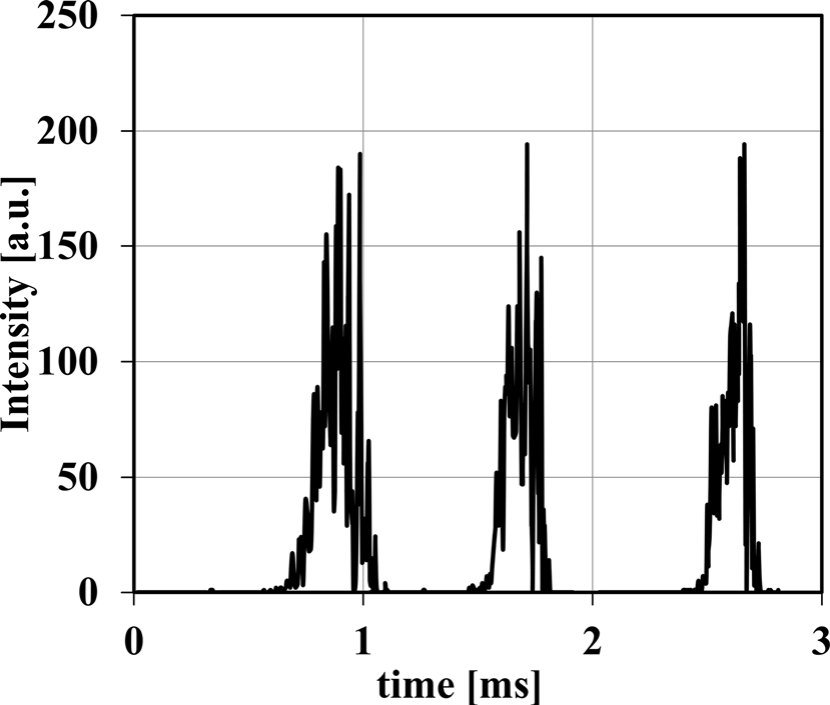
Sample of three different particle intensity profiles under the same plasma parameters (*I* = 500 A, $\dot{V}_{{{\text{gas}}}}$ = 120 slpm)

The key concern in this case is the fact that not all particles experience the same intensity profile, as the plasma jet fluctuates both in position and intensity, and small differences in particle trajectory (see CFD simulation flight paths in Fig. 4) can result in significantly different residence times and different penetration depths into the plasma jet. The intensity profiles shown in Fig. 10 result from three different particles which were overlaid onto the images of trial 9. The particles are shifted by 1 ms in the figure for an increased clarity. Even visually, it is apparent that there are significant differences in the intensities that these particles would experience traveling through the plasma jet. Consequently, the temperatures and velocities of the particles could vary likewise. Even for particles which follow the identical path, the differences can be significant, if they penetrate the plasma jet at an only slightly later point in time. This is visualized by a comparison of a particle flight path with intensities calculated with two different starting times, the second article starting 1 ms after the first. The visualization is available as supplementary material 4 and the associated link.

**Fig. 11:**
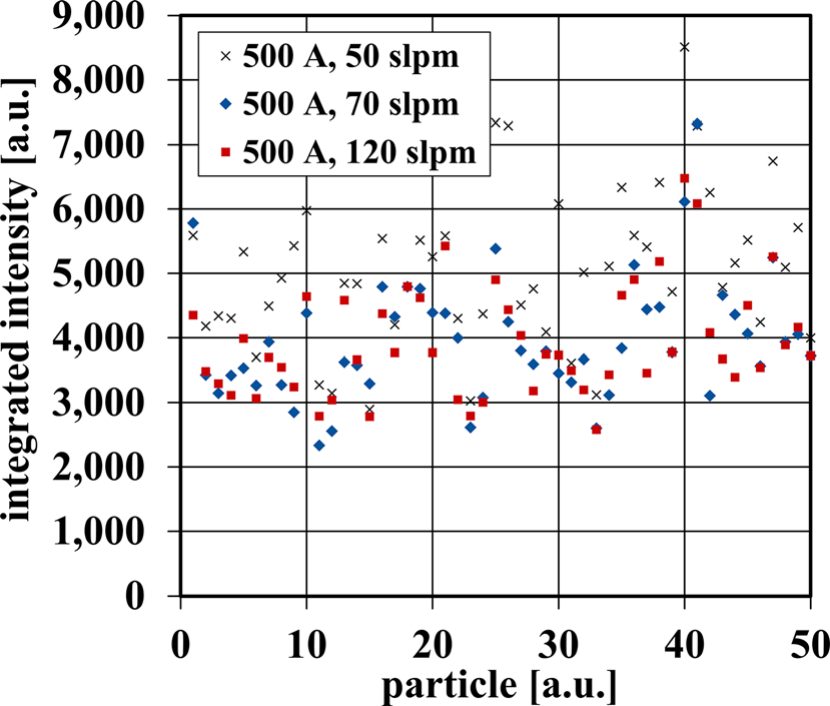
Total integrated intensities experienced by particles separated by process parameters (sample of 50 shown, data for 1000 were used in further analysis)

However, the fluctuation of intensities within the particle trajectories could be irrelevant if they result in the same particle properties like the temperature. It is easy to imagine that, for example, a moment of a particle in a very hot region of the plasma jet can be balanced again by a very cold one, resulting in no effect of the observed fluctuation. The particles are influenced over the entire path, therefore only the total absorbed energy from the plasma jet plays is important for its temperature. The same applies for its velocity. While the velocity cannot be considered with the here presented method, the temperature should correlate with the experienced intensity of the particles. Therefore, it seems reasonable to estimate the influence of the jet fluctuations onto the particles by integrating the particles’ intensity over its entire flight. Figure 11 allows us to compare the integrated intensities of 50 particles experienced for the different plasma jet conditions with an electric current of *I* = 500 A. It is obvious that there are strong variations between the integrated intensities of the individual particles. However, in this display, no statement can be made about the differences between the process parameters.

**Fig. 12:**
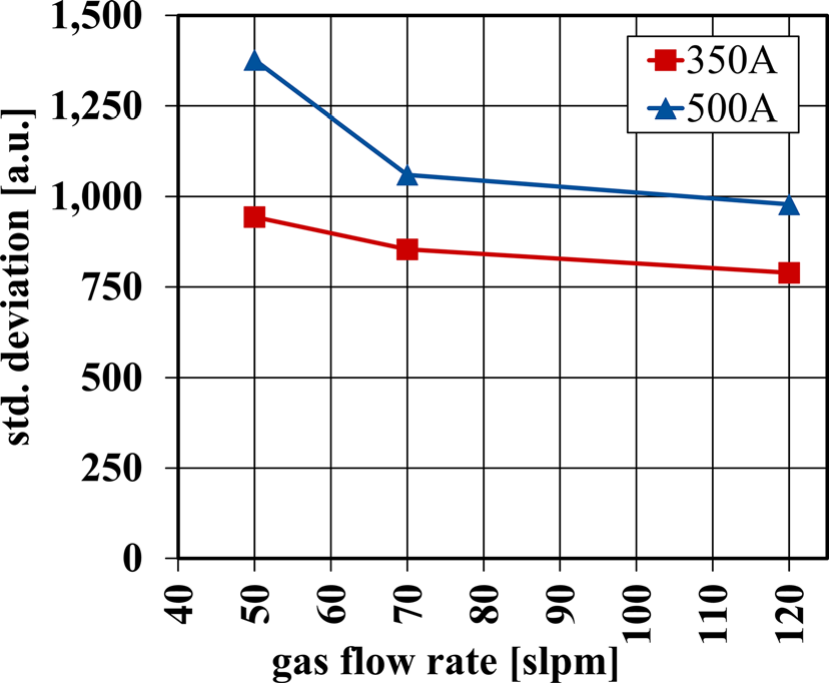
Standard deviation of integrated intensity for all particles under different current and gas flow parameters

To investigate this further the particle CFD simulation flight paths for all 1000 particles were applied to the plasma jet images with various parameters, as described previously. By analyzing the large number of particles, it is possible to carry out a statistical evaluation in order to assess the parameters quantitatively. As Fig. 11 shows, there is a large variance in the intensities experienced by different particles. While there are some trends through parameters, mentioned above, individual particles can receive twice as much intensity as others under identical process parameter conditions. The values of these integrated intensities are now statistically analyzed by calculating the standard deviation σ of the integrated intensities. This standard deviation is shown in Fig. 12 for the analyzed parameters. These were confined to parameter-specific groups in order to determine if certain parameters could impact the standard deviation, then this would reflect on which parameters improved the instability of the plasma jet and which worsened it.

Figure 12 illustrates the standard deviation of the total integrated intensities over time for the sprayed particles with different plasma jet parameters. It is clear that at higher gas flow rates the standard deviation of these intensities decreases. This would indicate a more stable process with the various particles experiencing more similar temperatures and heating more evenly. The change in *σ* when operating under different plasma jet parameters is particularly pronounced for the measurements with an arc current of *I* = 500 A. A similar, if less pronounced, trend is apparent for the electrical current of *I* = 350 A. The second observable trend is that as the current decreases, the standard deviation decreases as well. However, the trends observed here should not be overrated. Since the particle trajectories all originate from the simulation of the same parameter (*I* = 500 A, $\dot{V}_{{{\text{gas}}}}$ = 50 slpm), they are also only adjusted for this parameter. In order to target a comparison here, further trajectories must be calculated and mapped onto the recorded images.

## Conclusion

High-speed imaging proves to be a useful approach to analyze the plasma spraying process in detail. The fluctuations in the plasma jet are reduced by adjustments such as the implementation of cascaded electrode systems, however the particles still experience significant sudden plasma jet intensity fluctuation, in particular as they move in the jet toward the substrate and turbulence that cannot be avoided. This fluctuation is not considered if a plasma jet is assumed to heat homogeneously and not time dependently. By combining particle flight paths from a CFD simulation and high-speed imaging of a plasma jet under several operating conditions, the possible effect of such fluctuations was identified.

One key result of this investigation is that turbulence-induced fluctuations of plasma jet generated by the cascaded triple arc plasma generator, which is assumed to be stable, have the potential to affect the particle in-flight properties. The presented method has also shown that it will be able to detect differences between varying parameters in the future. With the help of further simulations, such investigations can be carried out in a next step. In addition, the results presented here still need to be further substantiated by investigations, such as measurements of particles’ in-flight properties.

## List of symbols

*C*_D_: Drag coefficient
CFD: Computational fluid dynamics
*d*_p_: Particle diameter
fps: Frames per second
*f*(*r*,*z*): Intensity of the 3D object at point in space
*F*(*r*,*z*): Intensity of the projection in the 2D plane
*I*: Arc current
$\dot{m}_{{\text{p}}}$: Powder feeding rate
*µ*_g_: Dynamic viscosity of the injector gas
Re_p_: Particles Reynolds number
*ρ*_g_: Density of the injector gas
*σ*_p_: Standard deviation of the particle distribution
$\dot{V}_{{{\text{gas}}}}$: Plasma gas flow rate
$\dot{V}_{{{\text{inj}}}}$: Injector gas flow rate
*v*_g_: Velocity of the gas
*v*_p_: Velocity of the particle
2D: Two-dimensional
3D: Three-dimensional
